# Unveiling belowground allelopathy: ^1^H-NMR spectroscopy reveals metabolic crosstalk and novel sterols in *Cyperus rotundus* and *Sorghum bicolor* co-cultures

**DOI:** 10.3389/fpls.2026.1715485

**Published:** 2026-02-04

**Authors:** Giulia Giorgi, Adriano Patriarca, Francesco Mura, Emma Cocco, Serena Taggiasco, Alessio Talone, Fabio Sciubba, Alfredo Miccheli, Alberta Tomassini, Walter Aureli, Daniela De Vita, Emanuele Zannini, Elisa Brasili

**Affiliations:** 1 Department of Environmental Biology, Sapienza University of Rome, Rome, Italy; 2 NMR-Based Metabolomics Laboratory (NMLab), Sapienza University of Rome, Rome, Italy; 3 R&D, Aureli Mario S.S. Agricola, Ortucchio, AQ, Italy

**Keywords:** ^1^H-NMR spectroscopy, allelopathy, *C. rotundus*, crops, root exudates, root-root co-culture

## Abstract

Root exudates are crucial for driving belowground ecosystem dynamics and subterranean chemical interactions. *Cyperus rotundus* (purple nutsedge) is a highly invasive weed, notoriously resistant to conventional control and causing significant crop losses globally. *Sorghum bicolor* is well-known for producing allelopathic compounds via root exudates. The metabolic shifts occurring during the direct interaction between *C. rotundus* and allelopathic crop *S. bicolor* are yet unexplored. In this study, we established an *in vitro* protocol for the isolation of root exudates from solid growth medium and employed a non-targeted ^1^H-NMR spectroscopy approach to fingerprint the hydrophilic and lipophilic root exudates within an *in vitro* co-culture system. A total of 21 metabolites, including carbohydrates, phenolics, peptides and fatty acids were identified. Notably, three new sterols including (3β)-3-hydroxy-6-methylstigmast-20(22)-en-14-oic acid, 6-methylstigmast-20(22)-en-14-oic acid derivative 1 and 6-methylstigmast-20(22)-en-14-oic acid derivative 2 were univocally assigned. Comparative analysis revealed that co-cultivation induced specific metabolic changes rather than a general increase in exudation. Specifically, the cyanogenic glycoside dhurrin, a primary allelochemical in *S. bicolor*, significantly decreased in co-cultures, while phytoalexins B, detected in individual *C. rotundus* exudates, was absent in co-cultures. However, no significant reduction in root biomass was observed for either species over the 12-day experimental period. These findings suggest that while root-root proximity triggers specific secondary metabolites shifts, these changes did not translate into immediate growth inhibition under the tested conditions. This study provides the first non-targeted metabolic profiling of the direct belowground interaction between *S. bicolor* and *C. rotundus* and highlights the complexity of modulating allelopathic responses in sterile environments.

## 1 Introduction

With agriculture facing escalating demands for food production alongside growing environmental concerns, the management of weeds has become a critical challenge. Weeds represent one of the major constraints to crop production, as they compete with crops for essential resources but also pose threats to food safety and agricultural sustainability due to the extensive reliance on herbicides, resulting in environmental contamination and the emergence of herbicide-resistant weeds (Parven et al., 2025). Moreover, some weed species harbor pests and diseases, acting as reservoirs for plant pathogens that can devastate crops (Chauhan, 2020). Climate change, with extreme temperatures and prolonged drought conditions, has significantly altered weed flora composition, favoring the proliferation of C4 weed species that exhibit higher photosynthetic efficiency, greater adaptability to stress and the ability to exploit environmental resources (Kumar et al., 2024). According to the urgent need to develop alternative and sustainable weed management strategies, allelopathy offers a promising and environmentally friendly tool in an integrated weed management plan. The biological phenomenon involves a chemical interaction between plants able to release allelochemicals with strong phytotoxic activity and the potential of being applied in agriculture to manage weeds (Radivojević et al., 2025). Allelochemicals, that are typically secondary metabolites, are released by leaching from leaves or litter on the ground, as volatile organic compounds from leaves, via residue decomposition and through root exudation (Kong et al., 2024). These compounds vary in the mechanism of action, uptake and effectiveness, and most of them are still unknown. *Cyperus rotundus* (L.), or purple nutsedge, belonging to the family *Cyperaceae*, is widely recognized as one of the most noxious weed species as it is highly invasive and it is able to avoid conventional control methods. It is a perennial sedge, which multiples rapidly through an extensive network of underground tubers having strong apical dominance, enabling its persistence across a wide range of environmental conditions (Sivapalan, 2013). This species inflicts high yield losses (20-90%) in over 50 crops worldwide (Peerzada, 2017). Up to now, no chemical or mechanical method has been effective in preventing its growth and spread. Among plant species with suppressive weed potential, *Sorghum bicolor* (L.) Moench stands out for its ability to produce a wide range of allelopathic compounds, including hydrophilic substances, phenolic acids and their aldehyde derivatives, as well as hydrophobic molecules such as sorgoleone from hydrophobic root exudates (Głąb et al., 2017). Several studies have suggested that its root exudates possess allelopathic activity, impairing the growth and competitiveness of neighboring plants and co-occurring or subsequent weed species in agroecosystems (Hussain et al., 2021; Krmanj and Kawa, 2021). Ranked as the fifth most important cereal crop globally, after wheat, maize, rice and barley (Espitia-Hernández et al., 2020), *S. bicolor* is primarily cultivated as a source of (gluten-free) grains, fodder, sugar and fiber, as well as for bioenergy production. It exhibits strong drought resistance, requires minimal input, and achieves high biomass yield (Badigannavar et al., 2018). *S. bicolor* was also proven to be effective in the suppression of *C. rotundus* when incorporated in the form of powder and extract in the culture medium (Chang et al., 2024), or when sprayed onto *C. rotundus* leaves in the form of an alcoholic or aqueous extract (Matos et al., 2020). However, to the best of our knowledge, no attempts have been made to demonstrate the effective allelopathic activity of *S. bicolor* root exudates in reducing the growth and fitness of *C. rotundus* when the two species interact. Root exudates comprise a wide array of primary and secondary metabolites released by plant root systems into the surrounding soil, where they serve essential ecological and physiological functions (Canarini et al., 2019). These metabolites include amino acids, phenolics, fatty acids, terpenoids, lipids, sugars, peptides, organic acids, enzymes, hormones, nucleotides, flavonoids, polyphenols, sterols, and volatile organic compounds (Salem et al., 2022; Upadhyay et al., 2022). By modulating the chemical and physical properties of the soil, root exudates influence the structure and function of the rhizosphere microbiome, ultimately contributing to optimized plant growth conditions (Chen and Liu, 2024). Root-root interactions between neighboring plants can influence the composition of root exudates. These exudates can, in turn, chemically affect the growth of surrounding plants through mechanisms such as allelopathy and allelobiosis, exerting either negative or positive effects by modulating resource competition, influencing plant development, and shaping the structure and dynamics of the rhizobiome (Kong et al., 2024; Zambelli et al., 2025). Despite increasing attention, the chemically driven mechanisms underlying root placement patterns and belowground signaling remain poorly understood. Elucidating the identity and roles of soil-borne signaling compounds could significantly advance our understanding of how root-mediated chemical communication influences the competitive ability and fitness of neighboring plants. Nevertheless, the analysis of root exudates presents several challenges due to the complexity of both the growth matrix and the chemical composition of the exudates themselves. A primary issue is that, most analytical approaches are often targeted, focusing on a single type or class of chemicals and relying on chromatography techniques. Given the huge structural diversity of the released compounds, it is challenging to obtain a comprehensive inventory of the root exudate metabolome using a single analytical method (Oburger and Jones, 2018; Dundek et al., 2011). In the present study, a non-targeted analysis of the root exudate metabolome isolated from a solid medium was performed by using ^1^H-NMR spectroscopy to investigate the dynamics of allelopathic interactions between *C. rotundus* and *S. bicolor.* Our central hypothesis was that co-culture conditions would trigger specific qualitative changes in the root exudate profiles, specifically regarding the synthesis and release of secondary metabolites involved in plant-plant signaling and defense, rather than a general quantitative increase in total exudate concentration. By comparing the metabolic profiles of individual and co-cultured root exudates, we aimed to identify the key chemical mediators, including both hydrophilic and lipophilic compounds, that characterize the competitive response between this highly invasive weed and the allelopathic crop.

## 2 Materials and methods

### 2.1 *C. rotundus* and *S. bicolor in vitro* co-culture growth conditions

Seeds from *S. bicolor* genotype SW2GS26 (Padana Sementi Elette S.r.l., Tombolo PD, Italy) were surface sterilized using 70% (*v/v*) ethanol under agitation for 1 min 30 sec, rinsed three times with dH_2_O and soaked in a solution of sodium hypochlorite (14 % Cl active chlorine) on a shaker SKO-D XL (ARGOlab) for 25 min at 300 rpm and then rinsed 5 times in sterile dH_2_O under a laminar flow hood. *C. rotundus* tubers were harvested from Aureli Mario S.S. Agricola company (Ortucchio, AQ, Italy) in the Fucino Plain (41°52′ N, 12°12′ E; 680 m AMSL). Tubers were surface sterilized by a first agitation on a shaker SKO-D XL (ARGOlab, Carpi MO Italy) in dH_2_O for 5 min at 300 rpm followed by 2 min in 70% (*v/v*) ethanol. After that, they were sonicated for 15 min in a solution of sodium hypochlorite (14 % Cl active chlorine) and 0.02% Tween, then rinsed 3 times with dH_2_O and subjected to another agitation step in a sodium hypochlorite solution (14 % Cl active chlorine) for 15 min before rinsing 5 times with sterile dH_2_O under a laminar flow hood. Every sterilization step was followed by a vigorous brushing of the tubers. Solid root co-cultures were established inoculating a seed from *S. bicolor* and a tuber from *C. rotundus* in magenta vessels with a rectangular base (11.4 × 8.6 × 10.2 cm) (Phytatray™ II, Sigma-Aldrich, Saint Louis, MO, USA), containing Murashige and Skoog (MS) salt medium (2.2 g/l), 1 % sucrose and 0.8 % agar, adjusted to pH 5.6–5.8. Culture medium was sterilized at 121°C for 20 min in an Autoclave Vapour Lite (VWR, Radnor, PA, USA). Sixteen co-cultures were prepared, with the two species placed approximately 1 cm apart. Fifteen cultures of a single *C. rotundus* tuber and 14 cultures of a single *S. bicolor* seed were used as control samples. From these, three independent biological replicates for co-cultures, *C. rotundus* and *S. bicolor* controls were used for the final NMR analysis and quantification. All cultures were grown in sterile Magenta vessels, which remained hermetically sealed for the entire duration of the experiment to prevent any external microbial contamination. The cultures were kept in growth chamber with a photoperiod (16 h light/8 h dark) for 12 days at 150 μE m−2s−1 intensity of white light and under controlled temperature (25°C light/24°C dark). To ensure the maintenance of sterile conditions throughout the 12-day growth period, vessels were sealed with micropore tape, and cultures were visually inspected daily for any signs of microbial growth. Only vessels showing no visual signs of contamination were processed for root exudate extraction.

### 2.2 Root exudates collection and extraction procedure

**Figure 1:**
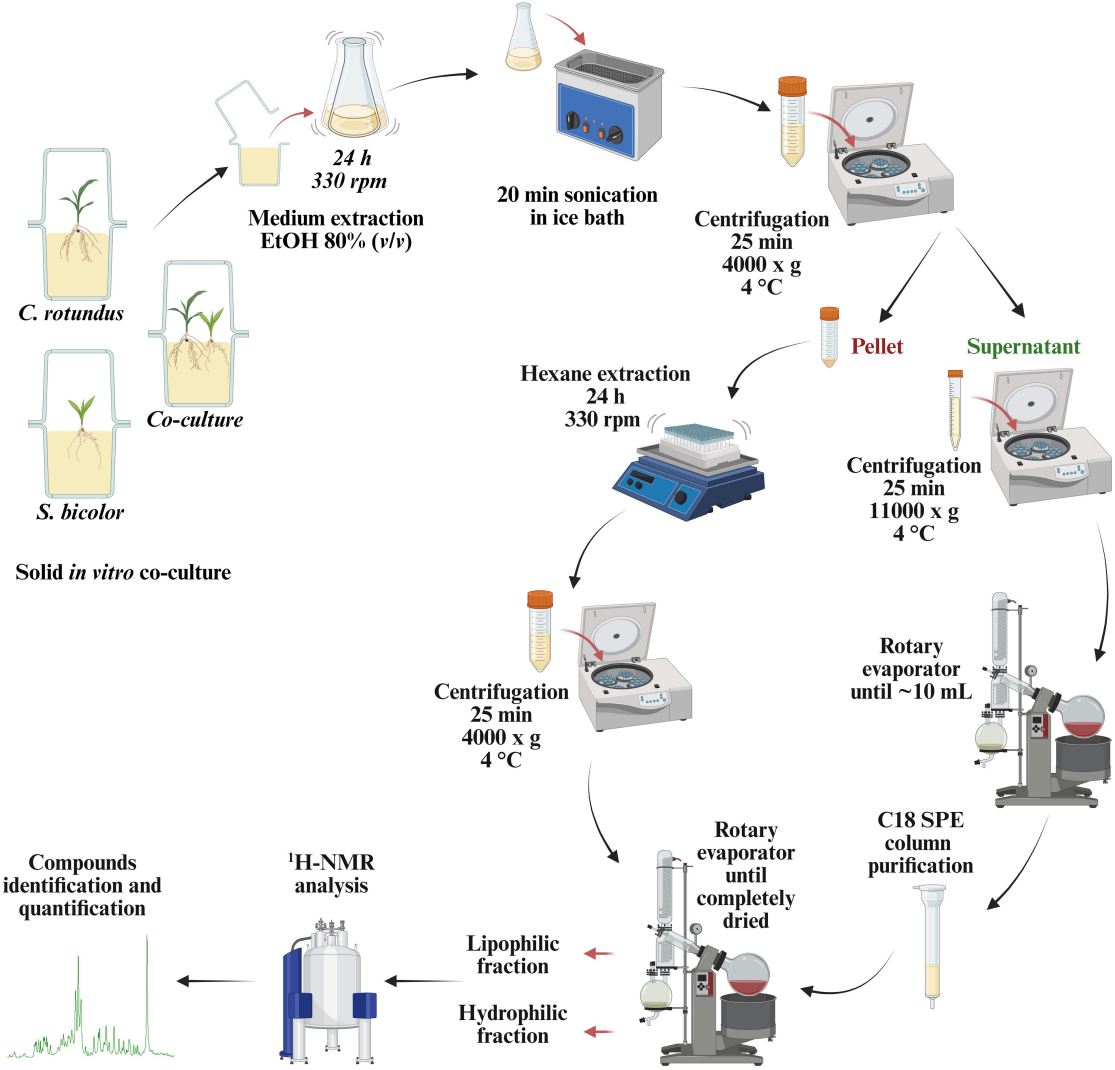
The analytical workflow: extraction and analysis procedure for the characterization of root exudates metabolome in solid medium.

Roots were carefully removed from the agar culture medium. Fresh root biomass was sampled and weighed. The agar was broken into small pieces and transferred into Erlenmeyer flasks. Plant’s roots and the magenta vessels were rinsed with 80 mL of ethanol [80% (*v/v*)] and the washes were added to the Erlenmeyer flasks, resulting in a 1:1 (*v/v*) ratio of agar to extraction solvent. The flasks were incubated at RT for 24 h on a Shaker SKO-D XL (ARGOlab) at 330 rpm. Flasks were sonicated in an ice bath for 20 min, then the culture medium from each culture container was divided in aliquots into 50 mL tubes and centrifuged for 25 min at 4 000 x g at 4°C. Supernatants were collected in 15 mL tubes and centrifuged for 25 min at 11000 x g at 4°C. While the agarized medium residuals were resuspended in hexane until fully submerged for 24 h on a Shaker SKO-D XL (ARGOlab) at 330 rpm. The supernatants were pooled and evaporated with a rotary evaporator (Buchi B-100 Heating Bath) until reaching a volume of ~10 mL. A C18 SPE column (SolEx C18) purification step was followed using 5 mL of methanol and 5 mL of MilliQ H_2_O 0.01% formic acid for column conditioning and 10 mL of MilliQ H_2_O with 0.01% formic acid for washing. Samples were then eluted in 5 mL of methanol/MilliQ H_2_O (80/20 *v/v*). The eluates were then transferred to vials and completely dried with a rotary evaporator. Tubes containing pellets in hexane were centrifuged for 25 min at 4000 x g at 4°C. Supernatants were collected and pooled into round-bottom flasks and completely dried with a rotary evaporator (Buchi R-100). Both hydrophilic and lipophilic fractions were stored at - 80°C until further analysis. Schematized work flow is presented in **Figure 1**.

### 2.3 ^1^H-NMR samples preparation and analysis

The hydrophilic phase was resuspended in 0.7 ml of D_2_O containing 3-(trimethylsilyl)-propionic-2,2,3,3-*d*_4_ acid sodium salt (TSP, 2 mM) as an internal and chemical shift reference standard, while the lipophilic phase was resuspended in 0.7 ml of CDCl_3_ containing hexamethyldisiloxane (HMDSO, 2 mM), as an internal and chemical shift reference standard. The hydrophilic and the lipophilic phases were then analyzed by ^1^H-NMR.

For the NMR Experiments was employed a JNM-ECZ 600R (JEOL Ltd., Tokyo, Japan) spectrometer operating at the proton frequency of 600 MHz and equipped with a multinuclear z-gradient inverse probe head, working at 298 K. For the hydrophilic phase monodimensional ^1^H experiments employed a presaturation pulse sequence for water suppression, using a time length of 2 seconds, a spectral width of 9.03KHz and 64k data points, corresponding to an acquisition time of 5.81 s. The pulse length of 90°flip angle was set to 8.3 μs, the recycle delay was set to 5.72 s. Similar parameters were employed for the lipophilic phase, without the presaturation sequence for water suppression.

Spectral assignment was carried out based on chemical shift, multiplicity, J couplings, ^1^H-^1^H TOCSY, ^1^H-^13^C HSQC and ^1^H-^13^C HMBC correlations, following the protocol reported by Sciubba et al., 2020 with the bidimensional experiments following spectral parameters in Spinelli et al., 2022. The putative assignments were corroborated by databases and literature compilations (Baskaran et al., 2022; Wishart et al., 2025).

Quantification of metabolites was done by comparing integrals of non-overlapping and univocally assigned resonances (bold resonances reported in **Supplementary Table S1**), with the integral of the respective internal standards (for TSP 9 protons in the aqueous fraction, for HMS 18 protons in the lipophilic fraction), according to the general formula:


$$C_{m}=\frac{A_{m}}{A_{IS}}\times \frac{H_{IS}}{H_{m}}\times C_{IS}$$

C_m_ stands for metabolite concentration, A_m_ is the area of the metabolite signal, A_IS_ is the area of the Internal Standard (IS) signal, H_IS_ is the number of protons generating the IS signal, H_m_ is the number of protons generating the metabolite signal, C_IS_ is the concentration of the IS. As per detection limit and reproducibility of metabolites quantified by NMR, for a JNM-ECZ 600R (JEOL Ltd., Tokyo, Japan) spectrometer operating at the proton frequency of 600 MHz, the limit of detection (LOD) is bound to micromoles. Regarding technical reproducibility, for multiple experiments done on the same sample, the uncertainty associated with the quantification is between 1 and 3% of the real value, also including the processing steps.

### 2.4 Statistical analysis

Statistical analysis was performed on MATLAB^®^ R2023a (MathWorks, Natick, Massachusetts, USA) with the Statistics and Machine Learning Toolbox package. Significant differences between sample groups were checked by one-way ANOVA test. Prior to this test, normality and homoscedasticity of each variable distribution was tested with The Shapiro-Wilk and the Brown-Forsythe test (Brown and Forsythe, 1974), with a significance value of 0.05. If these conditions were not met, a non-parametric ANOVA test was carried out with Kruskal-Wallis (Stevens, 2007). For the ANOVA-positive variables, with Bonferroni (Peluso et al., 2021). All pairwise multiple comparison tests were applied to determine which categories were discriminated by these metabolites (p< 0.05).

## 3 Results

The analysis of root biomass did not reveal significant differences in either plants (*S. bicolor* and *C. rotundus)* when grown in co-cultures compared to their respective single plant controls. Specifically, the average root biomass measured was approximately 12.4 ± 2.5 mg for *S. bicolor* and 9.1 ± 3.1 mg for *C. rotundus*. These values remained consistent in co-culture conditions, respectively 12.7 ± 2.7 and 7.2 ± 1.9 mg.

**Figure 2:**
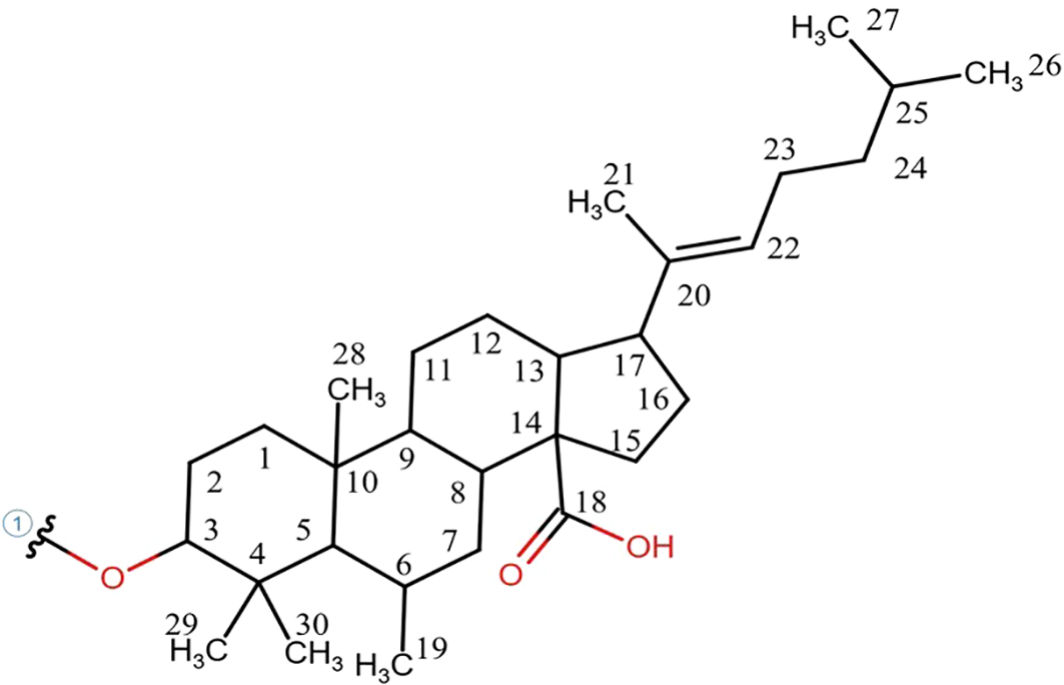
Molecule structure of the novel-sterol compound observed in ^1^H-NMR lipophilic spectrum of the exudate of *C. rotundus* and *S. bicolor* roots. The main structure corresponds to (3β)-3-hydroxy-6-methylstigmast-20(22)-en-14-oic acid, the other two sterols identified were assigned as 6-methylstigmast-20(22)-en-14-oic acid derivative 1 and 6-methylstigmast-20(22)-en-14-oic acid derivative 2, due to the missing C3 correlations.

**Figure 3:**
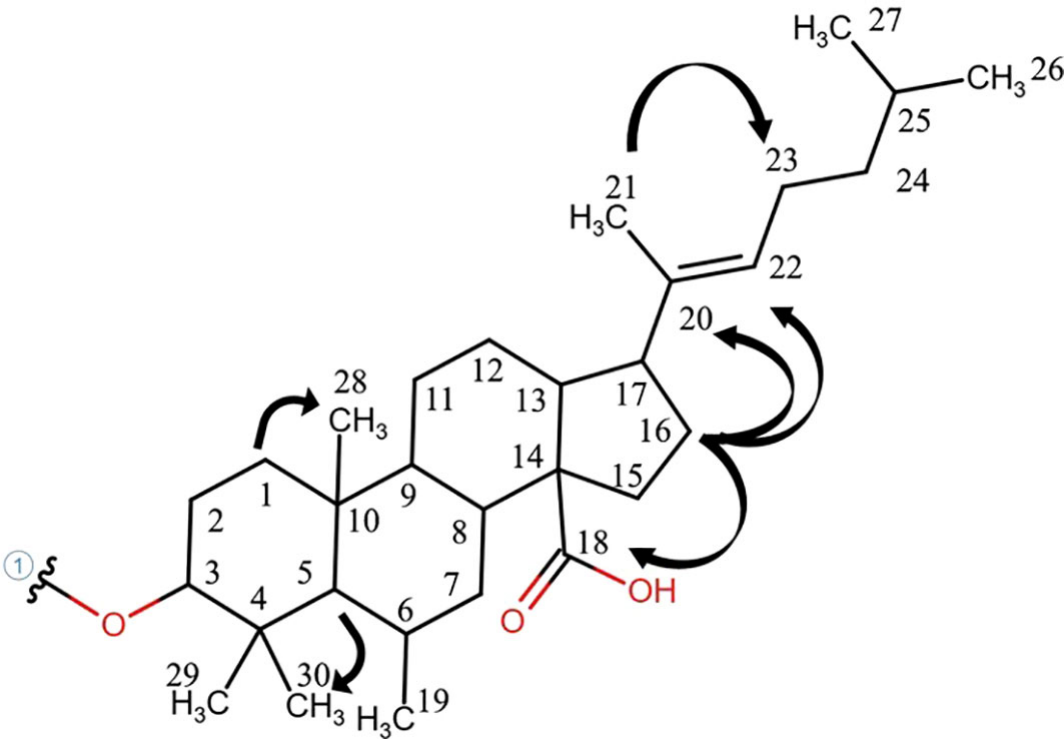
Key HMBC correlations of the novel-sterol compound, observed in ^1^H NMR lipophilic spectrum of the exudate of *C. rotundus*.

The ^1^H-NMR analysis of root exudates obtained from *C. rotundus* and *S. bicolor* co-cultures allowed the identification of 21 compounds (**Supplementary Table S1**), including carbohydrates, phenolic compounds, peptides, organic acids, fatty acids, terpenoid compounds and lipids. In particular, root exudates from co-cultures were rich in branched, linear, and aromatic peptide-like structures, acetic acid, trimethylamine, glucose, lupeol, monoacylglycerols, 7-hydroxycoumarin, and 4-methylbenzaldehyde (**Supplementary Table S1**). Three new sterols, named (3β)-3-hydroxy-6-methylstigmast-20(22)-en-14-oic acid, 6-methylstigmast-20(22)-en-14-oic acid derivative 1 and 6-methylstigmast-20(22)-en-14-oic acid derivative 2 were assigned and quantified. Their structure and resonances are reported in **Figures 2**, **3**, **Supplementary Table S2**. The 6-methylstigmast-20(22)-en-14-oic acid derivative 1 and 6-methylstigmast-20(22)-en-14-oic acid derivative 2 were putatively assigned due to similar resonances in C5 and C22 to (3β)-3-hydroxy-6-methylstigmast-20(22)-en-14-oic acid, but definitive HMBC correlations could not be verified due to low sensibility. Additionally, a series of unidentified metabolites (labeled as “U”) were detected, although a definitive structural assignment was not possible. However, their spectral patterns closely resemble those reported in the literature, suggesting the presence of Phytoalexin B (U01, U02) and xanthurenic acid (U03) in root exudates.

**Figure 4:**
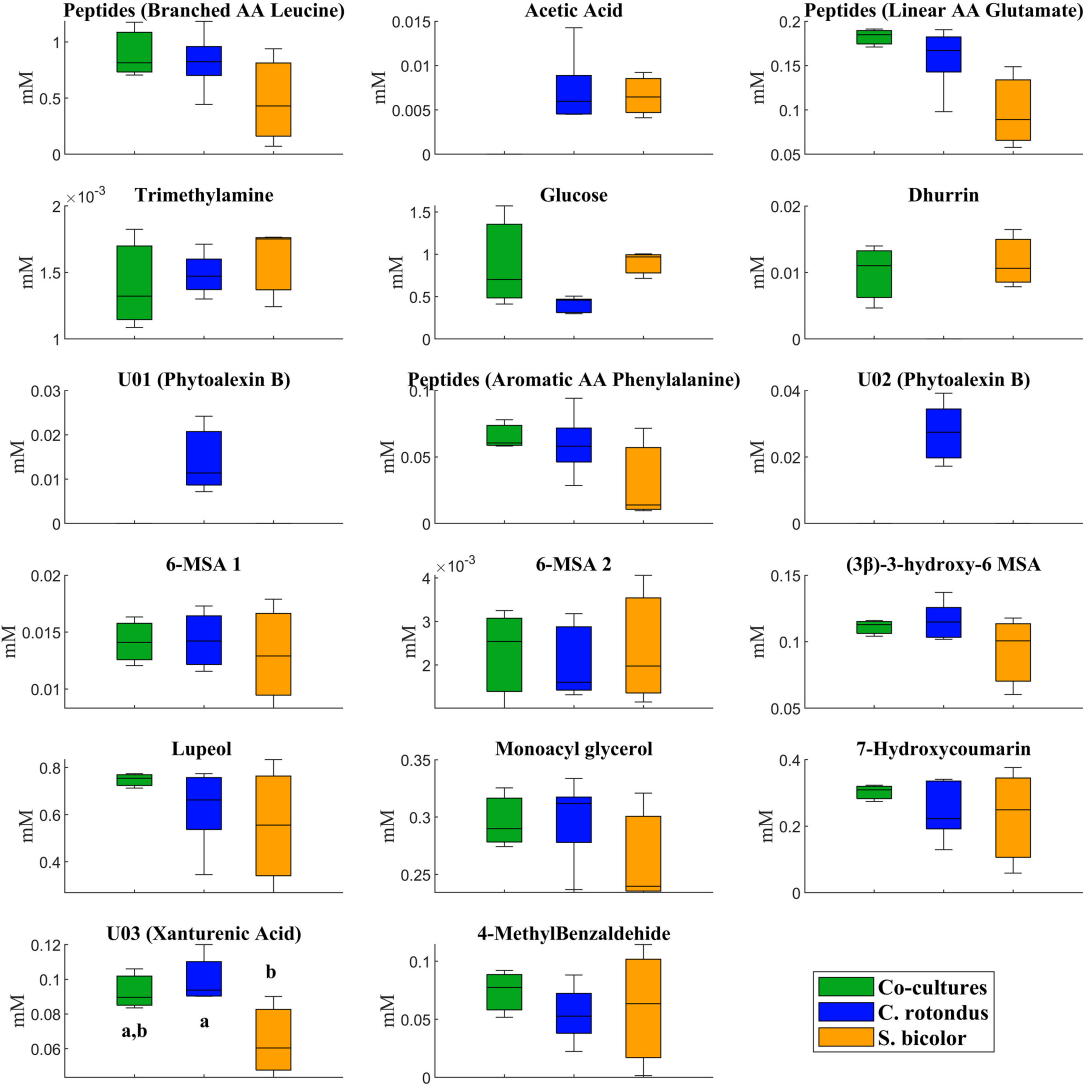
Boxplot of metabolites identified and quantified in co-cultures and individual cultures of *C. rotundus* and *S. bicolor*. Data is expressed as mM for three biological samples. Different letters, when reported, indicate statistical significance between groups at p<0.05 as revealed by ANOVA/Kruskal-Wallis tests with Bonferroni *post-hoc* analysis. 6-MSA stands for 6- methylstigmast-20(22)-en-14-oic acid.

The concentration of metabolites identified and quantified in both co-cultures and single cultures of *C. rotundus* and *S. bicolor*, is reported in **Figure 4**.

Some metabolites were found exclusively in individual cultures. Among them, U01 (Phytoalexin B) and U02 (Phytoalexin B) were detected in *C. rotundus* exudates but not in co-cultures, while dhurrin was present in *S. bicolor* exudates and significantly decreased in co-cultures (**Figure 4**), as well as acetic acid that was present in both individual cultures but not in co-cultures. U03 metabolite (xanturenic acid) was significantly higher in *C. rotundus* compared to *S. bicolor*, while in co-culture conditions reverted to levels comparable to those observed in the *C. rotundus*.

## 4 Discussion

This study was designed to investigate the complex allelopathic interactions between *C. rotundus* and *S. bicolor* by characterizing their root exudate metabolome within an *in vitro* co-culture system. The pressing global need for sustainable weed management, especially against pernicious invasive species like *C. rotundus*, underscores the importance of deciphering the underlying chemical mechanisms of plant-plant interactions. The application of ^1^H-NMR spectroscopy for non-targeted metabolomic profiling of root exudates provides crucial insights into these chemical communications moving beyond the limitations of targeted analysis. The ^1^H-NMR analysis led to the identification of 21 compounds in root exudates including carbohydrates, phenolic compounds, peptides, organic acids, fatty acids, terpenoids, and lipids. The major achievement of this study is the assignment and identification of three new sterols, belonging to the pentacyclic triterpene ester pathways: lupeol, (3β)-3-hydroxy-6-methylstigmast-20(22)-en-14-oic acid, and 6-methylstigmast-20(22)-en-14-oic acid derivative 1 and 6-methylstigmast-20(22)-en-14-oic acid derivative 2 from the root exudates of *S. bicolor* and *C. rotundus* (Yamashita et al., 2019). Pentacyclic triterpenoids, most present in *C. rotundus* root exudates, represent natural secondary metabolites, consisting of six isoprene units and characterized by a closed pentacyclic ring structure. In plants, these compounds are mainly synthesized via the mevalonate (MVA) pathway, in which squalene is first converted into 2,3-oxidosqualene by squalene epoxidase. The subsequent cyclization of 2,3-oxidosqualene, catalyzed by oxidosqualene cyclases (OSCs), leads to the formation of four main pentacyclic triterpene skeletons: α-amyrin, β-amyrin, lupeol, and friedelin (Li et al., 2023). Pentacyclic triterpenoids play important roles in interspecies communication, defense responses, and sensory regulation (Li et al., 2023). Beyond their ecological roles, pentacyclic triterpenoids are also valued for their bioactive properties and utilized in functional foods, pharmaceuticals, and industrial applications (Wang et al., 2021; Jäger et al., 2009). These compounds have been shown to have significant therapeutic and tissue-protective effects, including anti-tumor, hepatoprotective, and glucose-regulating effects, among others (Teng et al., 2024). Previous studies have shown allelopathic effects of *Alstonia scholaris* pentacyclic triterpenoids, such as betulinic acid, oleanolic acid, and ursolic acid on *Bidens pilosa* growth, a weed found growing abundantly near *A. scholaris* forests (Wang et al., 2014). El Ayeb-Zakhama et al. (2015) showed that the ethyl acetate extracts rich in pentacyclic triterpene acids as oleanolic acid, ursolic acid and corosolic acid from *Citharexylum* sp*inosum* L. flowers were phytotoxic to germination and seedlings growth of radish (*Raphenus sativus* L.), lettuce (*Lactuca sativa* L.) and canary grass (*Phalaris canariensis* L.) (El Ayeb-Zakhama et al., 2015). Pentacyclic triterpenoids are widely distributed in monocotyledonous plants, and the lupane-type compounds as well as the oleanolic acid were already identified as antioxidant compounds in *C. rotundus* tubers (Kamala et al., 2018). Lupeol (Kakarla et al., 2016) and lupeol-derived compounds such as lup-12, 20 (29)-dien-3β-ol-3-α-L-arabinopyranosyl-2’-oleate (lupenyl 3β-O-arabinpyranosyl 2′-oleate) (Sultana et al., 2017), and lup-12, 20 (29)-dien-3β-ol-3-α-L-arabinofuranosyl-2’-octadec-9”-eonate (Singh and Sharma, 2015) have been previously reported in *C. rotundus* rhizomes. Lupeol is widely distributed across numerous plant families and is commonly found in a variety of medicinal plants and traditional herbal remedies. It exhibits a broad spectrum of pharmacological activities, including anti-inflammatory, antitumor, antioxidative and antimicrobial effects (Liu et al., 2021). Beyond its medicinal relevance, lupeol also plays a role in plant physiology; for instance, it has been shown to negatively regulate root nodulation in *Lotus japonicus* and to influence nitrogen fixation activity (Lu et al., 2025). Furthermore, in an *in vitro* cultivation experiment with *Brassica nigra*, the application of lupeol as a salinity stress-mitigating agent demonstrated its capacity to protect plants from oxidative damage and to modulate cellular redox balance under stress conditions (Zia et al., 2023). Given the identification of lupeol and its derivatives among the root exudates of both species, it is plausible that these pentacyclic triterpenoid esters may contribute to the modulation of the rhizosphere. By selectively attracting beneficial microorganisms, such compounds might support the plant’s competitive ability in co-cultivation systems, potentially influencing fitness, nutrient acquisition or stress tolerance (Huang et al., 2019). Triterpenoids have also been identified in *S. bicolor* grains depending on the stage of development (Palmer and Bowden, 1977). It is known that triterpenoids are also chemical constituents of epicuticular waxes, where they contribute to cuticle reinforcement and play a role in reducing water loss under high-temperature stress conditions (Busta et al., 2021). However, to date, there is no evidence supporting their occurrence in root tissues or their release as components of root exudates. The identification of these previously undescribed compounds suggests the existence of unique biosynthetic pathways into allelopathic interactions where sterols act as major modulators of plant- plant beyond plant-microbe interactions (Der et al., 2024). The study carried out by Der et al. (2024) emphasizes that plant plasma membranes (PMs) are highly enriched in phytosterols, which regulate PM biophysical properties, spatial organization, and permeability. The discovery of novel sterols in root exudates, which are in direct contact with the rhizosphere, suggests a potential role for these compounds not only in direct allelopathic effects but also in influencing soil organic matter both directly, by binding with soil minerals or persisting within aggregates, and indirectly, by shaping microbially mediated processes that influence soil organic carbon stocks (Couvillion et al., 2025). Hydrophobic substances like lipids in root exudates shape the assembly and activity of microbial communities, impact soil physical structure, induce soil respiration, and cause priming, decomposition, and mineralization of soil organic matter, while also playing a key role in soil organic carbon accumulation and persistence through organo-mineral associations and enhancing soil water repellency and aggregate stability (Zhalnina et al., 2018; De Vries et al., 2019; Sasse, 2023). While the specific function of these newly identified sterols remains to be elucidated, their presence raises intriguing questions about how they might influence neighboring plant PMs or the associated microbial communities, thereby indirectly affecting competitive outcomes. The observed changes in known metabolite concentrations between individual cultures and co-culture conditions indicate that *C. rotundus* was predominantly responsible for the excreted root compounds. In co-culture, metabolites levels largely reflected their individual contribution rather than an additive effect of both species. Indeed, Phytoalexin B (U01 and U02) was exclusively detected in individual *C. rotundus* exudates but not in co-culture. Phytoalexins are plant defense compounds, often induced by stress or pathogen attack. Their absence in the co-culture suggests that *S. bicolor*’s presence might modulate *C. rotundus* defense responses. This could imply that *S. bicolor* either directly inhibits the production of these phytoalexins, or that the competitive stress from *S. bicolor* does not trigger the same defense pathways in *C. rotundus* that are active in isolation. This could hint at a potential advantage for *S. bicolor* in the competitive interaction by influencing the chemical vulnerability of *C. rotundus.* The fluctuations in xanthurenic acid (U03) levels, with higher concentrations in individual *C. rotundus* and a return to *C. rotundus*-like levels in co-culture, further underscore the dynamic nature of these chemical interactions. This uncharacterized metabolite likely plays a role in the metabolic adjustments during plant-plant recognition and competition. On the other hand, the significant decrease of dhurrin in *S. bicolor* co-culture is particularly insightful. Dhurrin, a cyanogenic glycoside, is a well-known allelochemical produced by sorghum. Its reduced production when co-cultured with *C. rotundus* might indicate that *S. bicolor* reallocates resources towards other defense mechanisms or growth in response to competition. In addition, *C. rotundus* could emit chemical signals that specifically suppress dhurrin biosynthesis in *S. bicolor*, potentially as a competitive strategy. This aligns with Kong et al. (2024) concept that plants detect and recognize neighbors and initiate allelopathic interference or adjust their responses. The allelochemical 7-hydroxycoumarin, known for its role in plant-plant interactions, was consistently detected in the root exudates of both species, whether grown individually or in co-culture, without significant variations. Coumarins, a key class of phenolic compounds and a significant family of plant secondary metabolites, are well-established for their role in allelopathy (Stringlis et al., 2019). Coumarins have been shown to delay seed germination by reducing endogenous gibberellin (GA4) levels and, consequently, the accumulation of Reactive Oxygen Species (ROS) (Chen et al., 2021). Their presence in the co-culture system therefore likely suggests a contribution to the observed allelopathic dynamics. Furthermore, 7-hydroxycoumarin can undergo various biochemical modifications—such as hydroxylation, glycosylation, or methylation—to produce derivatives like esculin or scopoletin. These transformations may significantly influence the compound’s bioactivity and persistence within the rhizosphere (Hijazin et al., 2019). Despite the novel insights provided by our study, some limitations must be acknowledged. The use of an *in vitro* solid growth medium allowed for a precise, non-targeted characterization of root exudates without the interference of soil organic matter. However, these conditions do not fully replicate the complexity of natural soil environments. In the field, metabolite dynamics are influenced by soil structure, pH, and, most importantly, the native microbial community, which can rapidly degrade or transform plant-emitted compounds. Therefore, the allelopathic interactions observed here represent a simplified model of the potential subterranean chemical communication.

## 5 Conclusion

Modern agriculture faces complex challenges, from increasing food demands to environmental concerns stemming from conventional weed management. Our study underscores the urgent need for sustainable alternatives to herbicides, particularly given the global threat posed by highly invasive, herbicide-resistant weeds like *C. rotundus*. Allelopathy, the chemical communication between plants, offers a promising eco-friendly strategy, and *S. bicolor* stands out for its allelopathic potential. Although *S. bicolor* did not significantly inhibit the growth of *C. rotundus*, the obtained results offer new insights by demonstrating the direct allelopathic interactions between both species through their root exudates in a controlled *in vitro* co-culture system. The ^1^H-NMR spectroscopy allowed us to characterize a diverse array of metabolites released by both species and to identify three unreported sterols, whose presence and roles in root exudates were entirely unknown. Our findings also revealed significant shifts in the profile of known allelochemicals, such as the decreased presence of dhurrin from *S. bicolor* and the exclusive detection of phytoalexin B in individual *C. rotundus* cultures. Moreover, the prevalence of pentacyclic triterpenoids, particularly lupeol and its derivatives, primarily from *C. rotundus* exudates, highlights their likely significant role in rhizosphere modulation, potentially influencing microbial communities and enhancing plant fitness or stress tolerance. The identification of these novel and changing metabolite profiles provides a deeper understanding of the complex chemical signals exchanged in belowground environments confirming the multifaceted roles of root exudates in shaping the soil environment and interspecific interactions. Future research should prioritize the functional validation of the newly identified sterols and other significant metabolites to confirm their specific phytotoxic or allelobiosis-inducing effects. Moving from controlled *in vitro* systems to soil-based experiments will be essential to verify the stability and bioactivity of these allelochemicals in real agricultural settings.

## Data Availability

The original contributions presented in the study are included in the article/**Supplementary Material**. Further inquiries can be directed to the corresponding author.
