# Supplementary material for: Unveiling belowground allelopathy: 1H-NMR spectroscopy reveals metabolic crosstalk and novel sterols in Cyperus rotundus and Sorghum bicolor co-cultures
Source: Front Plant Sci. 2026 Feb 4;17:1715485. doi: 10.3389/fpls.2026.1715485 (PMC12913420; doi:10.3389/fpls.2026.1715485)
Supplement: Supplementary file 1 [file Table1.docx]

Supplementary Material

**New insights into belowground allelopathic dynamics between *Cyperus rotundus* and *Sorghum bicolor* roots as revealed by ^1^H-NMR spectroscopy**

Giulia Giorgi^1§^, Adriano Patriarca^1§^, Francesco Mura^1^, Emma Cocco^1^, Serena Taggiasco^1^, Alessio Talone^1^, Fabio Sciubba^1,2^, Alfredo Miccheli^2,3^, Alberta Tomassini^3^, Walter Aureli^3^, Daniela De Vita^1^, Emanuele Zannini^1^ and Elisa Brasili^1,2^

^1^ Department of Environmental Biology, Sapienza University of Rome, Rome, Italy,

^2^ NMR-Based Metabolomics Laboratory (NMLab), Sapienza University of Rome, Rome, Italy

^3^ R&D, Aureli Mario S.S. Agricola, Via Mario Aureli 7, 67050 Ortucchio (Aq), Italy; produzione@aurelimario.com

**Supplementary Table 1**: Metabolites identified in the ^1^H NMR spectrum of the exudate of *C. rotundus* and *S. bicolor* roots. In bold are evidenced the resonances observed in all samples for each group; s: singlet, d: doublet, t: triplet, q: quadruplet, dd: doublet of doublets, m: multiplet, bs: broad signal, /: not observed or <LOD.  U01, U02 and U03 are putative assignations of molecules whose HSQC and HMBC correlation could not be detected.

| **Compound** | **^1^H δ ppm** | **^13^C δ ppm** | **Multiplicity** | **Assignment** |
| --- | --- | --- | --- | --- |
| **Peptides (Branched AA Leucine)** | **0.84-1.01**  1.28  1.47  1.56-1.68  2.08  4.15 | **24.0**    27.14   27.14  40.01   38.77   56.63 | **bs**   m   m   m   m   m | **δ,δ’-CH_3_**  ℽ’-CH  ℽ’’-CH  ℽ-CH, β-CH_2_  β-CH  α-CH |
| **Acetic Acid** | **2.05** | **25.9** | s | **CH_3_** |
| **Peptides (Linear AA Glutamate)** | **2.36**  1.59  4.30 | **36.76**  29.72   58.88 | **bs**   m   m | ℽ-CH_2_  **β-CH_2_**  α-CH |
| **Trimethylamine** | **2.89** | **47.4** | s | **N- CH_3_** |
| **Glucose** | **5.23**  **4.69**  3.55  3.72  3.42  3.84  3.73,3.90 | **98.66**  **94.83**  62.0-63.3  73.9-78.8   70.6-70.7   72.6-74.3  75.26-61.8 | **d**  **d**  m  m  m  m  m | **CH-1 α-Glucose**  **CH-1 β-Glucose**  CH-2  CH-3  CH-4  CH-5  CH_2_-6 |
| **Dhurrin** | **5.98**  6.99  7.52 | **73.65**  111.81  124.74 | **s**  d  d | **CH-2**  CH-2,6 (ar)  CH-3,5 (ar) |
| **U01 (Phytoalexin B)** | **6.68-6.70**  7.47  7.52 | **/**  **/**  **/** | **m**  m  m | **/**  **/**  **/** |
| **Peptides (Aromatic AA Phenylalanine)** | **7.22-7.45** | **130-132** | bs | **CH 2-6** |
| **U02 (Phytoalexin B)** | **8.07**  7.69  7.54  6.56 | **/**  **/**  **/**  **/** | **m**  m  m  m | **/**  **/**  **/**  **/** |
| Choline | 3.21  3.51  4.07 | 56.61  70.14  58.22 | s  t  t | N-(CH_3_)_3_  CH_2_  CH_2_ |
| Fumaric Acid | 6.51 | 138 | s | CH=CH |
| Mesaconic Acid | 1.90  6.50 | 18.12   132.26 | s  s | CH_3_  CH |
| **Lupeol** | **2.66**  1.68  0.96 | **33.5**  25.30  26.00 | t  m  m | **CH**  CH  CH_2_ |
| **Monoacylglycerol** | **3.65-3,55**  4.05-4.15  3.82 | **61.23**  70.33  73.19 | **dd**  dd  m | **CH_2_**  CH_2_  CH |
| **7-Hydroxycoumarin** | **6.59**  7.07  7.29  7.32  7.79 | **115.32**   123.53   118.81  129.01   129.74 | **d**  dd  m  m  d | **CH-1**  CH-4  CH-6   CH-3   CH-2 |
| **U03 (Xanturenic Acid)** | **6.95**  7.17  7.40 | **/**  **/**  **/** | **t**  m  m | /  /  / |
| **4-MethylBenzaldehide** | **7.80**  6.97  9.97 | **132.12**  131.01  192.12 | **d**  d  **s** | CH 2,6  CH 3,4  CH |

**Supplementary Table 2.** Resonance assignation of the novel-sterol compound, labeled as Sterol 3, observed in ^1^H NMR lipophilic spectrum of the exudate of *C. rotundus* and *S. bicolor* roots*.* R- group corresponds to a hydrogen. Multiplicity for reference, s: singlet, d: doublet, t: triplet, q: quadruplet, dd: doublet of doublets, m: multiplet, bs: broad signal, /: not observed, * for non clearly observable resonances due to strong signal overlapping. In bold are reported the resonances chosen for quantification. For sterols 1,2 the same resonance was chosen for quantification, present at 0.56 and 0.64 ppm.

| **Position** | **^1^H ppm** | **Multiplicity** | **^13^C ppm** |
| --- | --- | --- | --- |
| 1 | 1.70 | m | 37.01 |
| 2 | 2.05 | m | 37/45 |
| 3 | 4.64 | m | 70.01 |
| 4 | / | / | 57.00 |
| **5** | **0.61** | **d** | **27.12** |
| 6 | 1.51 | m | 28.81 |
| 7 | 1.10 | m | 33.45 |
| 8 | 1.39 | m | 31.43 |
| 9 | 1.01-1.41 * | / | / |
| 10 | / | / | 45.63 |
| 11, 12 | 1.27-1.30 | m | 20.04-24.38 |
| 13 | 1.01-1.41 * | / | / |
| 14 | / | / | 46.76 |
| 15 | 1.64 | m | 37.29 |
| 16 | 2.58 | m | 37.7 |
| 17 | 0.98 | m | 24.77 |
| 18 | / | / | 173.53 |
| 19 | 0.97 | d | 26.15 |
| 20 | / | / | 143.82 |
| 21 | 0.84 | s | 27.03 |
| 22 | 5.35 | t | 129.88 |
| 23 | 1.63 | m | 24.5 |
| 24 | 2.01 | m | 26.9 |
| 25 | 2.58 | m | 37.7 |
| 26, 27 | 1.30 | d | 22.07 |
| 28 | / | s | 25.50 |
| 29, 30 | / | s | 11.4 |
